# The Institute Session of 1889

**Published:** 1889-02

**Authors:** Pemberton Dudley

**Affiliations:** General Secretary


### THE INSTITUTE SESSION OF 1889.

EDITOR HOMŒOPATHIC PHYSICIAN:—As a further announcement respecting the Institute session of 1889, I have to report as follows:

The Bureau of Surgery has received assurances of aid from a number of our distinguished surgeons, and will present a series of papers on "Surgery of the Brain," including Cerebral Localization; Symptoms of Cerebral Tumor—its Diagnosis and Treatment; Abscess; Gunshot Wounds; Tumors of the Dura Mater; Compound and Depressed Fractures; Epilepsy from Fractures, and indications for Trephining.

The Bureau of Pædology has promise of active aid from several co-workers in that department, and are encouraged with prospects of a good report on "Preventive Medicine in Pædology."

The Bureau of Obstetrics is engaged on a Report, which will embrace nine papers, relating to "Puerperal Complications." All these papers are to be the work of well-known obstetricians.

Encouraging reports are being received from individual members of the Bureaus of Clinical Medicine, Sanitary Science, Ophthalmology and Gynæcology.

The Committee on Medical Education will present a careful Report, embodying the views and suggestions of its various members. There will be no separate Papers.

Notice is also given that as the chairman of the Committee on Pharmacy has resigned—involving also his withdrawal from the Committee on Organization of Provers' Clubs, the President has appointed as chairmen of these Committees, Drs. T. F. Allen, of New York, on the former, and C. Wesselhœft, of Boston, on the latter. Those having business with these Committees should note the change.

PEMBERTON DUDLEY,
*General Secretary.*

